# The alarming link between metabolic disease and sugar-sweetened beverage consumption

**DOI:** 10.12669/pjms.40.5.9256

**Published:** 2024

**Authors:** Shahreen Ansar Khan, Musarrat Riaz

**Affiliations:** 1 Shahreen Ansar Khan; 2 Musarrat Riaz, Baqai Institute of Diabetes and Endocrinology, Baqai Medical University, Karachi - Pakistan

We are writing to express our concerns about the growing prevalence of metabolic diseases and the insufficient knowledge among healthcare professionals (HCPs) regarding the detrimental impact of sugar-sweetened beverage consumption. Recent research has shed light on the urgency of addressing this issue for the well-being of our community.

Metabolic diseases, including obesity, Type-2 diabetes, and cardiovascular diseases, have reached epidemic proportions in recent years, taking a heavy toll on public health and healthcare systems. The rise in these conditions is closely associated with lifestyle factors, particularly the consumption of sugar-sweetened beverages. These drinks are laden with excessive amounts of added sugars and have been linked to weight gain, insulin resistance, and an increased risk of metabolic diseases.

A recent study as per International Diabetes Federation’s Atlas said “Pakistan is ranked 3rd highest globally with 33 million people living with diabetes in 2021. If no policy action is taken immediately, the number of people living with diabetes will increase to 62 million by 2045”. Studies showed that “reducing consumption of sugary drinks can save thousands of lives annually and save hospital expenditure. Increasing tax on sugary drinks which passes the price to consumers is an evidence-based strategy to reduce its consumption. Saudi Arabia and many other countries have adopted this strategy to successfully reduce its consumption and related diseases” (https://www.advocacyincubator.org/).

The inadequate awareness among HCPs about the risks posed by sugar-sweetened beverages is a significant concern. Given their role in guiding and educating patients, it is imperative that they possess up-to-date information on the adverse health effects of these beverages. The gap in knowledge underscores the pressing need for further education and training on this crucial matter.

To address this gap, we urge our healthcare institutions and professional bodies to prioritize ongoing training and education programs for HCPs. Moreover, public health initiatives must increase their efforts to raise awareness among the general population about the dangers associated with excessive consumption of these beverages.

Our community’s health and well-being depend on informed choices. It is incumbent upon us, as a responsible society, to tackle this issue head-on and equip both our healthcare professionals and the public with the knowledge and tools they need to make healthier choices. The evidence is clear: reducing sugar-sweetened beverage consumption is a crucial step in the fight against metabolic diseases.
